# Fecal microbiota transplantation: from empirical remedy to precision medicine

**DOI:** 10.3389/frmbi.2026.1863308

**Published:** 2026-06-17

**Authors:** Junsheng Zhao, Yiming Fan, Keda Yang, Hainv Gao

**Affiliations:** 1 Key Laboratory of Artificial Organs and Computational Medicine in Zhejiang Province, Shulan (Hangzhou) Hospital Affiliated to Shulan International Medical College, Zhejiang Shuren University, Hangzhou, China; 2 State Key Laboratory for Diagnosis and Treatment of Infectious Diseases, National Clinical Research Center for Infectious Diseases, National Medical Center for Infectious Diseases, Collaborative Innovation Center for Diagnosis and Treatment of Infectious Diseases, The First Affiliated Hospital, Zhejiang University School of Medicine, Hangzhou, China

**Keywords:** *Clostridioides difficile* infection, dysbiosis, fecal microbiota transplantation (FMT), gut microbiome, gut-brain axis, inflammatory bowel disease (IBD), live biotherapeutic products (LBPs), microbiome-based therapeutics

## Abstract

Fecal microbiota transplantation (FMT) has evolved from an empirical remedy for recurrent Clostridioides difficile infection (rCDI) into a foundational platform for precision microbiome-based therapeutics. This comprehensive review details FMT’s journey, analyzing its multifaceted mechanisms of action—including restoration of colonization resistance, metabolic reprogramming via short-chain fatty acids and bile acids, and profound immunomodulation—which extend far beyond simple microbial replacement. We critically evaluate its established, high efficacy in rCDI and its expanding, albeit more variable, applications across a wide spectrum of gastrointestinal diseases (such as inflammatory bowel disease, irritable bowel syndrome, and constipation), neurological disorders (including Parkinson’s and Alzheimer’s disease), metabolic conditions, autoimmune diseases, and oncology (particularly in modulating response to immune checkpoint inhibitors and treating graft-versus-host disease). The review further discusses the critical challenges of donor-recipient variability, safety, and the lack of standardized protocols that have driven the field’s technical evolution. This progression encompasses refined processing methods like washed microbiota transplantation (WMT), diverse delivery routes including oral capsules, and the exploration of non-bacterial components like bacteriophages through fecal filtrate transplantation (FVT). Ultimately, we highlight the field’s trajectory toward next-generation, defined live biotherapeutic products (LBPs) and engineered microbial consortia, aiming to transition from the complex “black box” of whole stool to safer, more consistent, and rationally designed precision therapies that target the specific dysbiotic networks underlying diverse human diseases.

## 1 Introduction

**Figure 1:**
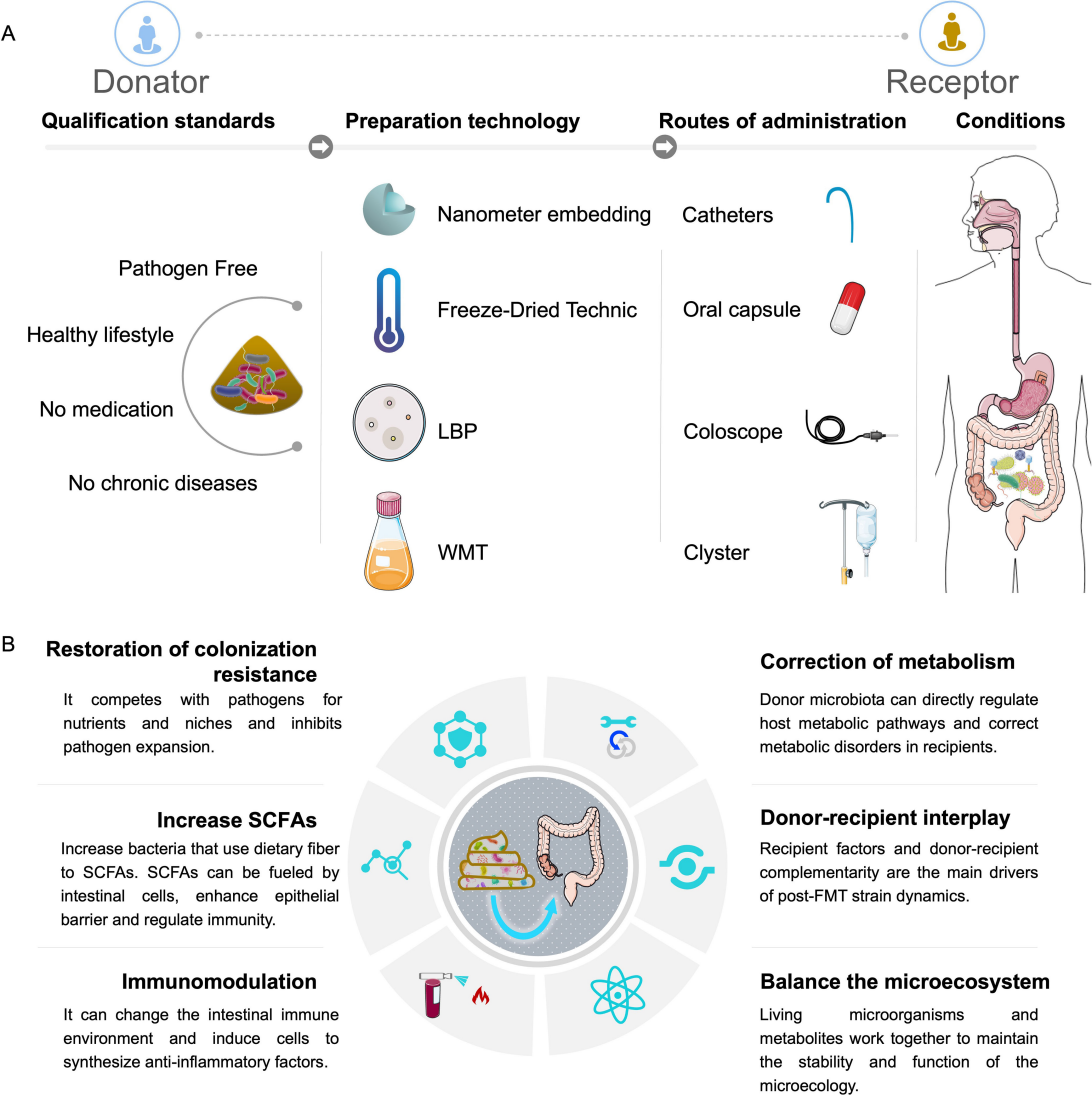
Overview of the process and principles of FMT. **(A)** The basic process of FMT. **(B)** Mechanism of FMT effect. FMT, fecal microbiota transplantation; LBP, Live biotherapeutic products; WMT, Washed microbiota transplantation; SCFA, short chain fatty acid.

The human gastrointestinal tract is colonized by a complex and dynamic ecosystem of microorganisms, collectively known as the gut microbiota, which plays a fundamental role in maintaining host health (Shao et al., 2023). This intricate community influences a vast array of physiological processes, from nutrient metabolism and immune system maturation to protection against pathogens (Liwinski and Elinav, 2020). Consequently, disruptions in the equilibrium of this ecosystem, termed dysbiosis, have been implicated in the pathogenesis of numerous diseases, ranging from gastrointestinal infections to systemic metabolic, neurological, and autoimmune disorders (Shao et al., 2023). The growing appreciation of the gut microbiota’s central role in human physiology and disease has catalyzed intense interest in developing therapeutic strategies aimed at its deliberate modulation (Young, 2016; Yadegar et al., 2024). Among these, fecal microbiota transplantation (FMT), defined as the transfer of processed stool material from a healthy donor into the gastrointestinal tract of a recipient, has emerged as the most direct and prominent “whole gut microbiome replacement” strategy (**Figure 1A**) (Yadegar et al., 2024).

Historically, the concept of using fecal material to treat disease dates back centuries, but its application remained sporadic and largely relegated to the fringes of medical practice. The modern resurgence of FMT was primarily driven by the clinical crisis of recurrent *Clostridioides difficile* infection (rCDI). Conventional antibiotic therapy, while effective for initial episodes, often fails to prevent debilitating recurrences by perpetuating the underlying microbial dysbiosis (Young, 2016; Maida et al., 2018). In this context, FMT demonstrated remarkable and consistent efficacy, effectively restoring colonization resistance and breaking the cycle of recurrence by reintroducing a diverse, healthy microbial community (Maida et al., 2018; Stallmach et al., 2020). This success propelled FMT from an anecdotal remedy into a rigorously studied intervention, leading to its integration into clinical practice guidelines for rCDI in many countries (Stallmach et al., 2020; Yadegar et al., 2024). The procedure proved to be not only highly effective but also largely safe when conducted with appropriate donor screening, establishing a crucial proof-of-concept for microbiome-targeted therapy (**Figure 1A**) (Stallmach et al., 2020).

However, the very nature of FMT—a complex, variable, and largely undefined biological product—introduces significant challenges as its application expands beyond rCDI (Yadegar et al., 2024; Wang et al., 2025c). The field is now in a critical transitional phase, moving from the initial “chaos” of empirical application toward an era demanding scientific precision (Wang et al., 2025c). While the efficacy in rCDI is well-established, the results of FMT in other conditions, such as inflammatory bowel disease (IBD), metabolic syndrome, and neurological disorders, have been variable and less predictable (Ghani et al., 2024; Yadegar et al., 2024). This variability underscores fundamental unanswered questions regarding FMT’s active therapeutic components, its precise mechanisms of action in different disease contexts, the optimal methods for donor selection and recipient matching, and the long-term safety and durability of its effects (Liwinski and Elinav, 2020; Yadegar et al., 2024; Wang et al., 2025c). The lack of standardized protocols for donor screening, preparation, and administration further complicates the clinical evaluation and broader adoption of FMT (**Table 1**) (Stallmach et al., 2020; Shao et al., 2023).

**Table 1:** FMT technology types and characteristics.

No.	Technology name	Description and definition	Processing method	Key advantages	Limitations or challenges
1	Conventional Fecal Microbiota Transplantation (FMT)	Transplantation of processed fecal material from healthy donors into the recipient’s gastrointestinal tract to reconstruct a healthy gut microbiota	Manually prepared crude fecal suspension, typically using fresh or frozen stool suspended in saline or other solutions, filtered to remove large particles	Low cost, relatively simple operation, well-established efficacy in rCDI	Psychological barriers exist, low standardization, potential presence of pro-inflammatory metabolites, viruses, or toxins, high inter-donor variability
2	Washed Microbiota Transplantation (WMT)	A refined FMT technique that removes supernatant through microfiltration and repeated centrifugation to obtain a high-purity microbial cell precipitate	Microfiltration + repeated centrifugal washing to remove soluble metabolites, viruses, endotoxins, and other non-cellular components, enabling precise dosing based on microbial cell count	Reduced adverse reactions, improved safety, facilitates standardization and quality control	High equipment and technical requirements, increased cost
3	Freeze-Dried Capsule FMT (cap-FMT)	Lyophilized fecal microbiota formulation encapsulated in enteric-coated capsules for oral administration	Stool samples undergo lyophilization, are ground into powder, and filled into enteric-coated capsules	Non-invasive, high patient compliance, convenient for storage and transportation, avoids risks associated with invasive procedures	Requires multiple capsules, risk of gastric acid degradation, reduced viability of certain bacterial strains
4	Fecal Filtrate Transplantation (FVT)	Transplantation of sterilized and filtered fecal supernatant containing phages, metabolites, and bacterial fragments, but no intact live bacteria	Fecal suspension filtered through a 0.22 μm membrane to remove all bacterial cells while retaining viral particles and small-molecule metabolites	Eliminates risk of bacterial transmission, suitable for severely immunocompromised individuals, higher safety profile	Therapeutic mechanisms not fully understood, long-term effects unknown
5	Live Biotherapeutic Products (LBPs)	Defined microbial combination therapy composed of single or multiple clonal bacteria with specific functions	Functionally characterized bacterial strains (e.g., VE303 containing eight Clostridium species) cultured in the laboratory and formulated at fixed ratios	Clear composition, reproducible manufacturing, compliant with pharmaceutical regulatory standards, controllable safety	Long development cycle, difficulty mimicking the complexity of natural microbiota
6	Anaerobically Prepared FMT	Processing and preservation of fecal samples under strict anaerobic conditions to maintain the viability of obligate anaerobic bacteria	Entire processing carried out in an anaerobic workstation to prevent oxygen exposure	Significantly improves survival and functional potential of key butyrate-producing bacteria such as *Faecalibacterium prausnitzii*	Requires specialized equipment, complex operation, high cost
7	Nano-Encapsulated FMT	Encapsulation of microbial cells using nanomaterials to enhance their survival and targeting within the gastrointestinal tract	Use of silk fibroin-phospholipid complexes or similar materials to encapsulate individual or consortia microbes into nanoparticles	Enhances microbial colonization efficiency and therapeutic efficacy, outperforms conventional FMT in animal models	Still in early research and development phase, not yet widely applied clinically
8	Autologous FMT (auto-FMT)	Patients store their own fecal material prior to antibiotic or chemotherapy treatment, followed by reinfusion to restore the original microbiota	Collection of feces during the individual’s healthy state, cryopreserved, then thawed and transplanted as needed	No immune rejection risk, eliminates transmission of foreign pathogens, highly personalized approach	Only applicable to patients who can pre-sample, not feasible for those with severe underlying diseases

This evolving landscape is being shaped by rapid advances in multi-omics technologies and bioinformatics, which are providing unprecedented insights into the composition and function of microbial communities (Yadegar et al., 2024). These tools offer the promise of moving beyond the crude transfer of undefined fecal matter toward a more refined understanding. Research is now focused on deciphering the specific microbial taxa, functional genes, viral components (virome), and metabolic products that mediate therapeutic benefits (Shao et al., 2023; Yadegar et al., 2024). The ultimate goal is to transition from FMT to next-generation, defined microbial consortia or precision microbiome-based therapeutics that can be consistently manufactured, rigorously tested, and tailored to specific patient populations and diseases (Ghani et al., 2024; Yadegar et al., 2024). This review sets the stage for a comprehensive exploration of FMT’s journey, examining its established foundations, its expanding frontiers, the technical and biological complexities it entails, and the path forward toward personalized, microbiota-based medicine.

## 2 Unraveling the mechanisms of action: how FMT exerts its therapeutic effects

The transition from an empirical procedure to a rational therapeutic intervention hinges on a deep understanding of how FMT works. While its clinical efficacy, particularly in rCDI, is well-established, the biological mechanisms mediating its success are multifaceted, interconnected, and vary across different disease contexts. Current research, empowered by multi-omics technologies, is moving beyond the simplistic concept of “replacing bad bugs with good ones” toward deciphering a complex interplay of microbial ecology, metabolic reprogramming, and host immunomodulation (Khoruts and Sadowsky, 2016; Porcari et al., 2023a; Andary et al., 2024).

A foundational mechanism, especially pertinent to CDI, is the restoration of colonization resistance—the ability of a healthy gut microbiota to prevent pathogen expansion (**Figure 1B**). This involves direct competition for nutrients and ecological niches, but a critical and well-characterized component is the reinstatement of a functional bile acid metabolism (Martinez-Gili et al., 2020; Khoruts et al., 2021; McMillan and Theriot, 2024). Antibiotic disruption depletes key bacterial taxa responsible for converting primary bile acids into secondary bile acids. Primary bile acids promote *C. difficile* germination, while secondary bile acids inhibit its vegetative growth and toxin production. FMT reintroduces the microbial consortia necessary to re-establish this metabolic conversion, thereby re-imposing a biochemical barrier against the pathogen (Martinez-Gili et al., 2020; McMillan and Theriot, 2024). This principle extends to other enteric pathogens, where FMT can restore a microbial community capable of outcompeting or inhibiting the growth of antibiotic-resistant organisms (Khoruts and Sadowsky, 2016).

Beyond direct pathogen inhibition, FMT profoundly reshapes the gut metabolome, with short-chain fatty acids (SCFAs) like butyrate, acetate, and propionate being key mediators. SCFAs, produced by bacterial fermentation of dietary fiber, serve as the primary energy source for colonocytes, strengthen the gut epithelial barrier, and possess potent immunoregulatory properties (Martinez-Gili et al., 2020; Tian et al., 2023; Andary et al., 2024). In conditions like IBD and rCDI, FMT has been shown to increase fecal SCFA levels, correlating with clinical improvement (Paramsothy et al., 2019; Martinez-Gili et al., 2020). Butyrate, in particular, can induce regulatory T cells and inhibit pro-inflammatory NF-κB signaling (Tian et al., 2023). Furthermore, SCFAs can directly influence neuronal and glial cell function. In a model of hypoganglionosis, FMT-enhanced SCFA production improved the efficacy of enteric neural crest cell transplantation by activating the MEK1/2 signaling pathway, highlighting a role in supporting tissue repair and neurogenesis (Tian et al., 2023).

Immunomodulation is a central pillar of FMT’s mechanism, particularly in immune-mediated diseases like IBD. Successful FMT alters the intestinal immune milieu, often shifting the balance from a pro-inflammatory to a regulatory or tolerant state (Burrello et al., 2018; Wu et al., 2023). In experimental colitis, therapeutic FMT was shown to control inflammation primarily by inducing interleukin-10 (IL-10) secretion from a broad array of immune cells, including CD4+ T cells, invariant natural killer T (iNKT) cells, and antigen-presenting cells (Burrello et al., 2018). Human studies in UC have mirrored these findings, with clinical response to FMT associated with increased systemic levels of the anti-inflammatory cytokine IL-10 and decreased levels of pro-inflammatory TNF-α and IL-6 (Huang et al., 2022a). This immunomodulatory effect can be strain-specific. For instance, the transfer of *Odoribacter splanchnicus*, a donor-derived bacterium coated with recipient immunoglobulin A (IgA) in responders, was found to increase colonic Foxp3+ regulatory T cells and IL-10 production, which was necessary for its protective effect in mouse models of colitis (Lima et al., 2022). In patients with fulminant CDI, the FMT microbiome rapidly modulates the systemic inflammatory response, subduing the systemic inflammatory storm (Khoruts et al., 2026). Similarly, in a Parkinson’s disease (PD) model, FMT’s neuroprotective effect was mediated through the suppression of systemic and neuroinflammation driven by the lipopolysaccharide-TLR4 signaling pathway (Zhao et al., 2021).

The microbial ecology of the procedure itself—the interplay between donor and recipient communities—is a critical determinant of therapeutic outcome (He et al., 2022; Schmidt et al., 2022; Porcari et al., 2023a). Engraftment, the successful colonization and persistence of donor-derived microbes in the recipient’s gut, is not a uniform process but is influenced by recipient factors, donor-recipient compatibility, and procedural variables (He et al., 2022; Schmidt et al., 2022). Recipients with a highly dysbiotic baseline microbiota, often characterized by low diversity and dominance by facultative anaerobes like Enterobacteriaceae (an enterotype dubbed RCPT/E), may offer less resistance to donor strain colonization (He et al., 2022). Conversely, the resilience of recipient-native strains can impede donor engraftment. Large-scale analyses have shown that recipient factors and donor-recipient complementarity are the main drivers of post-FMT strain dynamics, which can occur independently of clinical outcome but are predictable using ecological modeling (Schmidt et al., 2022). This underscores that FMT efficacy depends not just on what is administered but on how the recipient’s ecosystem receives and integrates the new microbial immigrants (Porcari et al., 2023a). New research has found that when gut microbes switch from “competitive” to “cooperative”, disease occurs (Corral López et al., 2026). An explicit metabolic mathematical model was used to simulate the dynamics of the microbiota and an Ecological Network Balance Index (ENBI) was developed for quantitative assessment. This theory and model not only provide theoretical support for the effectiveness of FMT, but also can be used as a marker for diagnosis and prediction of diseases, and provide new ideas for future precise intervention: to find targets that can cut off the key cross-feeding chain (such as specific flora combinations, phages, and small molecule inhibitors), and pull the network from “cooperation” to “competition” (Corral López et al., 2026).

Mechanistic insights also reveal that FMT’s impact extends beyond bacteria. The transfer includes viruses (phages), fungi, and eukaryotes, which contribute to community stability and function. For example, the commensal protozoan *Tritrichomonas musculis* was shown to attenuate CDI pathogenesis in mice by modulating host arginine-ornithine metabolism and immune responses, enhancing mucosal protection (Yang et al., 2024). This highlights the importance of considering the entire transferred ecosystem. Furthermore, the viability of transferred microbes is crucial. Preparation protocols exposed to ambient oxygen can disproportionately reduce the abundance of strict anaerobes critical for SCFA production, such as *Faecalibacterium prausnitzii*, thereby potentially compromising the functional capacity of the transplant (Papanicolas et al., 2019).

Finally, emerging evidence points to more systemic mechanisms. FMT can correct metabolic perturbations, such as bile acid malabsorption in IBD, which is associated with a better clinical response (Lu et al., 2025). It can also influence host physiology at distant sites through microbial metabolites. The choline trimethylamine-lyase (CutC) pathway in specific gut bacteria generates trimethylamine (TMA), which is converted by the host into the pro-thrombotic metabolite trimethylamine N-oxide (TMAO). Transplantation of microbes containing a functional *cutC* gene is sufficient to transmit enhanced platelet reactivity and thrombosis potential in gnotobiotic mice (Skye et al., 2018). This demonstrates how donor microbiota can directly modulate host metabolic pathways with systemic disease implications.

## 3 The established pillar: FMT for rCDI

While the systemic implications of FMT-mediated microbial metabolite transfer are a frontier of research, the therapy’s foundational and most robust clinical success remains in the treatment of rCDI (**Table 2**) (Vatkar et al., 2025). The disruption of gut microbiota by antibiotics, the primary risk factor for CDI, creates a rationale addressed by the restorative intent of FMT (Terveer et al., 2016; Yadegar et al., 2023). This application has evolved from a last-resort intervention to a well-established, guideline-recommended therapy, supported by extensive efficacy and safety data across diverse patient populations and clinical settings.

**Table 2:** Therapeutic efficacy and adverse events of FMT across different diseases.

No.	Disease category	Specific disease	Treatment efficacy	Adverse events and safety observations	Evidence level
1	Gastrointestinal Infections	Recurrent *Clostridioides difficile* Infection (rCDI)	RCTs show cure rates of 80%–90%, significantly superior to antibiotics; NNT = 3; can be considered a first-line option; effective even in patients with cirrhosis or post-transplant	Short-term AEs commonly include bloating and diarrhea, mostly mild and self-limiting; risk of MDRO transmission if screening is inadequate; long-term follow-up shows no significant increase in chronic disease risk	I
2	Inflammatory Bowel Disease	Ulcerative Colitis (UC)	Cochrane review indicates FMT increases clinical remission rate (RR = 1.79); RCTs show up to 32% steroid-free remission with colonoscopy plus enema delivery; efficacy linked to donor diversity and engraftment of butyrate-producing bacteria	Low risk of acute exacerbation; donor dependency and individual response variability observed in some studies; note higher FMT failure rates in active-phase IBD patients	I
3	Inflammatory Bowel Disease	Crohn’s Disease (CD)	Early trials suggest possible maintenance of remission; however, recent multicenter RCT was terminated early due to inefficacy; pediatric studies indicate oral capsules combined with nutritional support may improve inflammatory markers	Good safety profile; uncertain efficacy for inducing remission; potential resistance to FMT in patients positive for adherent-invasive *E. coli* (AIEC)	II
4	Inflammatory Bowel Disease (IBD)	Pouchitis	Case series report clinical remission in some patients; however, RCTs found no significant difference compared to placebo despite increased microbial similarity to donor	Safe and feasible; unstable efficacy, heavily influenced by factors such as antibiotic pretreatment	III
5	Functional Gastrointestinal Disorders	Irritable Bowel Syndrome (IBS)	Conflicting RCT results: some studies report 65% effectiveness with improved quality of life; others, including meta-analyses, find no significant overall symptom improvement, especially in oral capsule groups	Generally safe; high placebo effect complicates efficacy assessment; patient stratification by IBS subtype or baseline microbiota may identify responders	II
6	Functional Gastrointestinal Disorders	Chronic Constipation	RCTs confirm retrograde enema FMT in children significantly increases spontaneous bowel movement frequency; adult slow-transit constipation shows symptom improvement accompanied by microbiota and metabolic changes	Good safety profile; local irritation is the main short-term reaction	II
7	Hepatobiliary Diseases	Primary Sclerosing Cholangitis (PSC)	Preliminary studies show it is safe and feasible, increasing microbial diversity; reduction in alkaline phosphatase correlates with donor microbiota engraftment	Small-sample studies, long-term efficacy remains to be validated	IV
8	Hepatobiliary Diseases	Hepatic Encephalopathy (HE)	Can restore microbial balance, reduce ammonia and other neurotoxins, improve intestinal barrier function; clinical observations suggest improved cognition and reduced hospitalizations	FMT reverses neurotoxicity caused by phenylethylamine derived from *Ruminococcus gnavus*; good safety profile	IV
9	Hepatobiliary Diseases	Metabolic Associated Steatotic Liver Disease (MASLD)	WMT reduces hepatic steatosis, with mechanisms involving ILC3 cell homing to the liver	Clinical and preclinical studies support its potential, but larger-scale validation is still required	IV
10	Neurological Diseases	Parkinson’s Disease (PD)	RCTs demonstrate improvements in motor and non-motor symptoms (constipation, depression, anxiety), enhanced quality of life; recent trials show reduced α-synuclein aggregation	Most studies are small-scale; long-term neuroprotective effects require further confirmation	II
11	Neurological Diseases	Amyotrophic Lateral Sclerosis (ALS)	RCT did not significantly slow functional decline, but secondary endpoints showed improved constipation and mood symptoms, with sustained increases in *Bifidobacterium* levels	Safe and feasible, biological effects present; larger samples needed to verify clinical significance	II
12	Neurological Diseases	Alzheimer’s Disease (AD)	Currently exploratory stage, aiming to reduce neuroinflammation via gut-brain axis modulation and alter biomarkers	Insufficient clinical evidence, still in proof-of-concept phase	IV
13	Neurological Diseases	Anorexia Nervosa	Preliminary studies show oral FMT is feasible and capable of altering gut microbiota composition	Safety preliminarily confirmed; efficacy requires validation in subsequent trials	IV
14	Metabolic Diseases	Obesity/Metabolic Syndrome	Cochrane review deems evidence very low; limited BMI improvement in adolescents; other interventions show weak signals	More precise strategies needed; overall therapeutic efficacy currently unsatisfactory	IV
15	Metabolic Diseases	Type 1 Diabetes (T1D)	Discussed as a potential strategy, with diet-driven microbiota changes linked to pathogenesis, though human trial results are inconsistent	Efficacy remains unclear, mechanisms complex, requiring deeper investigation	V
16	Autoimmune Diseases	Atopic Dermatitis (AD)	RCTs show FMT significantly improves disease severity scores, associated with reductions in Th2/Th17 cells, IgE levels, and functional shifts in microbiota	Acceptable safety, suggesting immunomodulatory potential	IV
17	Autoimmune Diseases	Systemic Lupus Erythematosus (SLE)	Analyses suggest responders exhibit DNA hypermethylation, reversing disease-related hypomethylation, correlated with metabolites like caproic acid	Small-sample observational data; causality remains to be verified	IV
18	Autoimmune Diseases	Psoriatic Arthritis (PsA)	Single gastroscopy-guided FMT performed worse than sham procedure, with higher failure rate in FMT group and less improvement in disability scores	Indicates not all autoimmune diseases respond to FMT; potential negative effects may occur	IV
19	Oncology-Related Applications	Graft-versus-Host Disease (GVHD)	For steroid-refractory acute intestinal GVHD, prospective studies show most patients achieve complete remission, allowing tapering or discontinuation of immunosuppressants	Associated with restored microbial diversity, engraftment of butyrate-producing bacteria, and enhanced Treg responses; considered a breakthrough therapy	IV
20	Oncology-Related Applications	Immune Checkpoint Inhibitor (ICI) Sensitization	Phase I trials show high objective response rates with FMT plus anti-PD-1 in melanoma, prolonged survival; progression-free survival (PFS) also improved in NSCLC	Safe and feasible; heterogeneous efficacy, requiring optimization of donor selection and timing	IV
21	Oncology-Related Applications	Decolonization of MDRO	Safe and effective in immunocompromised patients, with high clearance rates; success positively correlated with greater microbiota dissimilarity between recipient and donor and extent of donor microbiota engraftment	Important strategy for preventing invasive infections, particularly suitable for patients before and after transplantation	IV
22	Aging-Related Conditions	Aging and Sarcopenia	FMT combined with resistance training improves muscle mass, function, and inflammatory markers, transferring a “healthy longevity” phenotype	Exploratory application, representing an emerging direction	V
23	Other Neurological Conditions	Stroke, Tourette Syndrome, Bipolar Disorder	Animal models show FMT provides protection or reverses cognitive deficits via SCFA restoration, 5-HT regulation, and other mechanisms	Lack of human evidence, still in preclinical stage	V
24	Other Ophthalmic Diseases	Glaucoma	Dysbiosis associated with glaucoma leads to decreased indole-3-acetic acid (IAA); FMT or IAA supplementation may exert neuroprotective effects	Emerging concept of the “gut-eye axis”, novel mechanism but distant from clinical translation	V

The clinical efficacy of FMT for rCDI is unequivocal. A systematic review of randomized controlled trials (RCTs) concluded that FMT leads to a large increase in the resolution of rCDI compared to alternative treatments like antibiotics, with a number needed to treat of three (Minkoff et al., 2023). This high efficacy is consistently reflected in real-world registries and cohort studies, reporting cure rates typically between 80% and 90% following a single procedure (Hagel et al., 2016; Kelly et al., 2021; Vaughn et al., 2023). A landmark RCT demonstrated the superiority of donor FMT over autologous transplant, with a clinical cure rate of 90.9% versus 62.5%, directly linking therapeutic success to the introduction of a healthy donor microbiota (Kelly et al., 2016). This efficacy translates into durable responses; a majority of patients maintain cure for at least one year even after exposure to traditional CDI risk factors like healthcare contact or acid suppressants, although post-FMT antibiotic use significantly undermines this durability (Saha et al., 2021a; Groenewegen et al., 2025).

The clinical application of FMT has been refined and expanded. Patients with fulminant CDI have a narrow treatment window, and their condition worsens, leaving perhaps only tens of hours for intervention. Research has successfully addressed practical barriers, demonstrating that freeze-dried, encapsulated FMT (cap-FMT) is as effective and safe as administration via colonoscopy, offering a convenient and scalable delivery method that avoids procedural risks (Youngster et al., 2016; Staley et al., 2017; Vaughn et al., 2023). Similarly, the use of frozen fecal preparations is non-inferior to fresh material, facilitating the establishment of stool banks for reliable, screened donor material (Lee et al., 2016; Terveer et al., 2016). These advances have supported the development of standardized, commercially prepared microbiota-based live biotherapeutic products (LBPs) (**Table 1**). Phase III trials of such products, administered via enema, have shown significant superiority over placebo in preventing rCDI recurrence (Khanna et al., 2022), and large open-label studies of FDA-approved rectal formulations confirm safety and efficacy in broad patient populations, including those with comorbidities like IBD (Feuerstadt et al., 2025).

The optimal timing of FMT is an area of ongoing evaluation. Current guidelines primarily recommend FMT for multiply recurrent CDI (Mullish et al., 2024). However, health-economic analyses suggest that FMT is a cost-effective strategy even at the first recurrence (Aby et al., 2022). Furthermore, evidence indicates that FMT can be a life-saving intervention in severe and severe-complicated CDI, dramatically reducing mortality and presenting a promising alternative to colectomy in certain patients (Fischer et al., 2017; Hocquart et al., 2018). A recent equivalence trial even suggests that FMT without antibiotic pretreatment may be considered non-inferior to vancomycin as first-line therapy for primary CDI, potentially expanding its role earlier in the disease course (Juul et al., 2025). Nonetheless, a randomized trial in patients who had responded to antibiotics for recurrent CDI found that subsequent cap-FMT did not reduce recurrence compared to placebo, highlighting that patient selection and treatment context remain critical (Drekonja et al., 2025).

FMT has proven effective in complex patient populations traditionally considered high-risk. In patients with cirrhosis, FMT is safe and achieves high cure rates for CDI, with failure associated with concurrent non-CDI antibiotic use and more advanced liver disease (Cheng et al., 2021). Solid organ transplant recipients with CDI also benefit from FMT, with high overall cure rates, although predictors of failure include severe disease and inpatient status (Cheng et al., 2019). Pediatric patients with rCDI show similarly high success rates to adults, though factors like fresh donor stool and colonoscopic delivery may be associated with better outcomes (Chen et al., 2017; Nicholson et al., 2020). The presence of concurrent IBD presents a unique scenario, as these patients are at higher risk for rCDI. A meta-analysis confirms FMT is effective for curing CDI in IBD patients, with overall cure rates up to 89% (Chen et al., 2018). However, single-center studies indicate that cure rates may be somewhat lower in IBD patients compared to those without IBD, and a failed FMT is often associated with clinically active IBD (Khoruts et al., 2016; Nicholson et al., 2022). Importantly, a significant proportion of IBD patients experience improvement in their underlying bowel disease activity following FMT for rCDI (Ianiro et al., 2021; Porcari et al., 2023b). Long-term data are reassuring, showing no intrinsic association between a history of multiply recurrent CDI or its treatment with FMT and the subsequent development of most cardiometabolic or immune-mediated conditions, though one claims-based analysis noted an increased incidence of myocardial infarction post-FMT requiring further study (Saha et al., 2021b; Dawwas et al., 2022). Prospective registry data with years of follow-up report a low risk of new medical conditions deemed related to FMT (Kelly et al., 2021; Yau et al., 2024).

The mechanisms underpinning FMT’s success in rCDI extend beyond simple diversity restoration. Engraftment of donor microbiota leads to durable compositional changes, including an increase in Clostridium clusters IV and XIVa and a normalization of bile acid profiles, with a gradual shift from primary to secondary bile acids that inhibit *C. difficile* germination (Jalanka et al., 2016; Chen et al., 2023; Yadegar et al., 2023). Furthermore, successful FMT is associated with functional alterations in the host, such as the upregulation of specific circulating microRNAs that were suppressed during active CDI, which may exert cytoprotective and immunomodulatory effects (Monaghan et al., 2021). Interestingly, the transfer of sterile fecal filtrates containing bacterial debris, proteins, and bacteriophages—but not intact living bacteria—has been shown to resolve rCDI in small studies, indicating that non-viable components or viruses can mediate therapeutic effects (Ott et al., 2017). This principle of microbial ecosystem restoration also underlies the exploratory use of FMT for decolonizing the gut of multidrug-resistant organisms (MDROs), with early studies showing promising eradication rates (Saha et al., 2019; Seong et al., 2020).

## 4 Frontiers in gastrointestinal diseases: FMT for IBD and beyond

Building on the paradigm established for rCDI, the exploration of FMT has extended into the realm of IBD, a chronic inflammatory condition of the gastrointestinal tract encompassing ulcerative colitis (UC) and Crohn’s disease (CD) (Imdad et al., 2023; Nagayama et al., 2025). The rationale is grounded in the well-documented dysbiosis—an altered composition and function of the gut microbiota—observed in IBD patients, which is believed to contribute to the aberrant immune response and mucosal inflammation characteristic of these diseases (Cammarota et al., 2015; Glassner et al., 2020). The goal of FMT in this context is not merely to displace a pathogen, as in rCDI, but to fundamentally remodel a dysbiotic ecosystem toward a state that supports immune homeostasis and mucosal healing (Pigneur and Sokol, 2016).

For UC, the most extensive clinical data exist. Systematic reviews concluded that FMT may increase the proportion of patients with active UC achieving clinical remission (Imdad et al., 2023; Ikram et al., 2026). This conclusion is supported by several RCTs. A pivotal Australian trial demonstrated that a short course of anaerobically prepared, pooled donor FMT delivered via colonoscopy followed by enemas resulted in a significantly higher rate of steroid-free clinical and endoscopic remission at 8 weeks compared to autologous FMT (32% vs 9%) (Costello et al., 2019). Similarly, a multicenter trial of intensive, multidonor FMT (administered via initial colonoscopic infusion followed by enemas five times weekly for 8 weeks) showed a primary outcome of steroid-free clinical remission with endoscopic remission or response in 27% of the FMT group versus 8% in the placebo group (Paramsothy et al., 2017a). These trials suggest that the FMT protocol, including preparation method (anaerobic), donor strategy (pooled/multidonor), and intensity of dosing, is critical for efficacy. Mechanistic insights from these studies indicate that clinical response is associated with increased microbial diversity post-FMT and the engraftment of specific donor-derived bacterial taxa and metabolic pathways, such as SCFA producers like *Eubacterium hallii* and *Roseburia inulivorans*, while the presence of *Fusobacterium* spp. was linked to non-response (Paramsothy et al., 2017a, Paramsothy et al., 2019). Further research has identified specific transferable strains, such as immunoglobulin A-coated *Odoribacter splanchnicus*, which in mouse models was shown to induce regulatory T cells and interleukin-10 production, limiting colitis (Lima et al., 2022). The importance of donor-recipient matching is increasingly recognized; studies indicate that clinical response may be donor-dependent and associated with the transfer and engraftment of specific beneficial strains rather than overall microbial engraftment (Van et al., 2024; Ishikawa et al., 2025). Furthermore, the recipient’s native microbiota significantly influences outcomes, with pre-FMT microbial profiles and the retention of certain native bacterial genera predicting treatment responsiveness (Rees et al., 2022; Zhao et al., 2025).

The evidence for FMT in CD is less consistent and more preliminary. An early pilot RCT in patients with colonic or ileo-colonic CD in remission suggested a higher steroid-free clinical remission rate at 10 and 24 weeks in the FMT group compared to sham transplantation, alongside a reduction in endoscopic disease severity (Sokol et al., 2020). However, the primary endpoint of donor microbiota engraftment was not met. A more recent multicenter RCT aimed at inducing remission in mild-to-moderate CD was terminated early for futility, finding no difference in combined clinical and endoscopic remission at 8 weeks between FMT and placebo groups (Kao et al., 2024). This highlights the potential distinction between using FMT for maintenance versus induction of remission in CD and underscores the need for optimized protocols. Studies suggest that successful outcomes may be linked to the engraftment of specific donor clades, such as Actinobacteria, and the loss of Proteobacteria, and that strain-level analysis is crucial for understanding FMT dynamics in CD (Kong et al., 2020). In pediatric populations, open-label studies have reported that oral FMT capsules combined with partial enteral nutrition can reduce inflammatory markers and enrich beneficial core functional genera like *Faecalibacterium* and *Roseburia* (Zou et al., 2025). Beyond conventional UC and CD, FMT has been explored in pouchitis, a common complication after ileal pouch-anal anastomosis. Case series and pilot studies have shown promising results, with some patients achieving clinical remission after FMT from a donor with a healthy pouch (Zaman et al., 2024; Kousgaard et al., 2025). However, a subsequent randomized, double-blinded, placebo-controlled trial found that multidonor FMT was comparable to placebo in inducing clinical remission, though it did increase donor microbiome similarity in recipients (Kousgaard et al., 2024). The variability in pouchitis study outcomes mirrors the broader challenges in IBD-FMT research, where factors like antibiotic pre-treatment, donor selection, and baseline disease activity significantly modulate results (Sarrabayrouse et al., 2020; Raja et al., 2025).

The application of FMT extends beyond classical IBD to other gastrointestinal disorders where dysbiosis is implicated. In irritable bowel syndrome (IBS), results from RCTs have been conflicting. A notable double-blind trial from Norway reported that 65% of patients receiving FMT via colonoscopy were responders at 3 months, compared to 43% in the placebo group, with benefits in quality of life and fatigue extending to 6 months (Johnsen et al., 2018, Johnsen et al., 2020). Another RCT focusing on IBS with predominant abdominal bloating found that a single nasojejunal donor FMT led to a higher response rate at 12 weeks compared to autologous FMT (56% vs 26%), with response associated with higher baseline microbial diversity (Holvoet et al., 2021). Conversely, a systematic review and meta-analysis of earlier RCTs found no significant benefit of FMT for global IBSs at 12 weeks, noting significant heterogeneity and a high placebo response, particularly in trials using oral capsules (Xu et al., 2019). This underscores that route of administration, donor material, and patient stratification (e.g., by baseline microbiome or IBS subtype) are likely critical determinants of efficacy (Shaukat and Brenner, 2019). In chronic constipation, FMT has shown therapeutic potential. A randomized trial in children with intractable constipation found that retrograde colonic enema-based FMT significantly increased the rate of spontaneous bowel movements compared to placebo enemas (Gu et al., 2024). In adults with slow transit constipation, FMT improved symptoms and was associated with shifts in gut microbiota and metabolites linked to protein digestion and absorption pathways (Xie et al., 2021).

Emerging research points to FMT’s potential in hepatobiliary and iatrogenic gastrointestinal conditions. A pilot study in primary sclerosing cholangitis (PSC), often associated with IBD, demonstrated that FMT was safe and led to increased microbial diversity; a decrease in alkaline phosphatase levels correlated with donor bacterial engraftment (Allegretti et al., 2019). Furthermore, FMT has been investigated as a mitigator of intestinal damage induced by chemotherapy or antibiotics. In experimental models, antibiotic-induced dysbiosis prior to chemotherapy exacerbated intestinal mucositis and impaired mucosal recovery, effects that were reversed by autologous FMT (Wardill et al., 2021). Similarly, FMT from berberine-treated rats ameliorated 5-fluorouracil-induced intestinal mucositis in recipient animals, mediated through modifications in gut microbiota and metabolites like butyrate (Chen et al., 2020). This highlights a potential adjunctive role for microbiota-based therapies in oncology supportive care.

Despite these explorations, the transition of FMT into routine clinical practice for IBD and other gastrointestinal diseases faces significant hurdles. The therapeutic effect is heterogeneous, and current research is focused on identifying predictors of response. Key factors under investigation include the donor’s microbial profile (e.g., high diversity, abundance of *Clostridiales* clusters and *Bacteroides*) (Rees et al., 2022), recipient factors (younger age, less severe disease, specific baseline microbiota) (Rees et al., 2022; Zhao et al., 2025), and the degree of donor microbiota engraftment (Sokol et al., 2020; Lima et al., 2022). The choice of administration route (lower GI via colonoscopy/enema often favored over upper GI for UC) (Paramsothy et al., 2017b), the need for antibiotic preconditioning (Strati et al., 2021; Raja et al., 2025), and the potential synergy with dietary interventions are active areas of optimization (Sarbagili et al., 2022). Notably, the presence of certain pathobionts, such as adherent-invasive *Escherichia coli* (AIEC) in CD, has been associated with severe mucosal dysbiosis and may create a resistant microbial environment that impedes successful donor engraftment post-FMT (Zhilu et al., 2021). This complexity has spurred the concept of precision microbiota therapy, moving beyond undefined donor stools toward rationally designed bacterial consortia, next-generation probiotics, or engineered microbial strains tailored to correct specific functional deficiencies in the recipient’s ecosystem (Ihekweazu et al., 2019; Nagayama et al., 2025; Quiroga-Centeno et al., 2025).

## 5 Beyond the gut: FMT in neurological, metabolic, autoimmune, and oncological disorders

The exploration of FMT has rapidly expanded beyond the confines of the gastrointestinal tract, driven by the growing recognition of the gut microbiota’s role in systemic physiology via axes such as the gut-brain, gut-liver, and gut-immune axes.

The neurological domain has become a major frontier for FMT research, with PD at the forefront. Preclinical models have demonstrated that FMT can correct rotenone-induced dysbiosis, ameliorate motor deficits, and suppress neuroinflammation and dopaminergic neuron loss, potentially via attenuating the lipopolysaccharide (LPS)-TLR4 signaling pathway along the microbiota-gut-brain axis (Zhao et al., 2021). This preclinical promise is increasingly supported by clinical trials. RCTs in mild-to-moderate PD patients have reported that FMT, administered orally or via colonoscopy, can lead to significant improvements in both motor and non-motor symptoms, including constipation, depression, anxiety, and quality of life (Cheng et al., 2023; Wang et al., 2025a). A recent phase 2 trial in drug-naïve PD patients further strengthened the evidence, showing that repeated donor FMT significantly improved motor symptoms and constipation, correlating with reduced colonic α-synuclein aggregation and increased fecal dopamine metabolites (Zhang et al., 2026). For amyotrophic lateral sclerosis (ALS), a double-blind RCT found that FMT did not significantly slow the decline in the primary functional score over 35 weeks (Feng et al., 2024). However, secondary analyses revealed benefits in constipation and mood symptoms, alongside a sustained increase in beneficial *Bifidobacterium*, suggesting a nuanced biological effect warranting larger trials (Feng et al., 2024). The potential of FMT extends to other neuropsychiatric conditions. In Alzheimer’s disease, modulation of the gut-brain axis via interventions like FMT is being explored as a strategy to reduce neuroinflammation and modify disease biomarkers, though clinical evidence remains limited (Lista et al., 2025; Sharma et al., 2025). A pilot study in females with anorexia nervosa found oral FMT to be feasible and capable of altering gut microbiota composition, setting the stage for efficacy trials (Panah et al., 2026). In animal models, FMT has shown protective effects in conditions ranging from cerebral ischemic stroke, via restoration of SCFA-producing bacteria (Chen et al., 2019), to Tourette syndrome, potentially through modulation of serotonin secretion (Li et al., 2022). Furthermore, gut dysbiosis linked to cognitive impairment in bipolar disorder was transferable via FMT to mice, inducing cognitive and synaptic deficits that could be partially reversed by healthy microbiota supplementation (Tang et al., 2025). As gut microbiota biological relevance to psychiatric disorders, FMT has also emerged as a potential treatment option (Yang et al., 2026). Anxiety/depression in IBS patients can also be transferred to mice via FMT, and *A. shahii* supplementation can reverse emotional symptoms in mice, constructed the *A. shahii*-indole-AhR pathway complete causal evidence chain (Wang et al., 2026). A novel gut-eye axis is also emerging, with evidence that glaucoma-associated dysbiosis reduces levels of the microbial metabolite indoleacetic acid (IAA), and that FMT or IAA administration can exert neuroprotective effects in models of the disease (Wang et al., 2025b).

In metabolic and hepatic disorders, FMT is investigated as a means to reshape a dysbiotic ecosystem implicated in disease pathogenesis. The historical interest in FMT for metabolic syndrome has evolved into more targeted applications (de et al., 2017). For metabolic dysfunction-associated steatotic liver disease (MASLD), clinical and preclinical studies indicate that washed microbiota transplantation (WMT, a refined FMT method) can reduce hepatic steatosis (Zhong et al., 2024). This effect may be mediated by promoting the homing of interleukin-22-producing group 3 innate lymphoid cells (ILC3s) to the liver via the CXCL16/CXCR6 axis (Zhong et al., 2024). In the more severe complication of liver cirrhosis, hepatic encephalopathy (HE) has been a primary target. FMT appears to restore microbial diversity, rebalance beneficial and pathogenic bacteria, reduce gut-derived toxins like ammonia, and improve intestinal barrier function (Bloom et al., 2021; Fang and Fang, 2025). A mechanistic study identified *Ruminococcus gnavus*-derived phenylethylamine (PEA) as a key neurotoxin in HE; FMT from HE patients to germ-free cirrhotic mice replicated neurological symptoms, underscoring the causative role of dysbiosis (He et al., 2025). Clinical observations suggest FMT can improve cognitive function and reduce hospitalizations in HE patients (Fang and Fang, 2025). FMT is also being explored in other contexts like alcohol-associated liver disease as a novel therapeutic strategy (Mullish and Thursz, 2024). Beyond hepatology, research touches on obesity and diabetes. A Cochrane review concluded that evidence for FMT in managing childhood and adolescent obesity is currently of very low certainty, showing little to no effect on outcomes like BMI in adolescents, though some signals exist for other microbiome-based interventions in broader age groups (Fahim et al., 2025). In type 1 diabetes, diet-driven microbiome alterations are implicated in disease risk, and while FMT is discussed as a potential therapeutic strategy, human trial data remain inconsistent (Girdhar et al., 2026). The intersection of microbiota with aging is also notable, with FMT studied as a tool to transfer “healthy longevity” and ameliorate age-related conditions like sarcopenia, where it combined with resistance training improved muscle mass, function, and inflammatory markers (Novelle et al., 2025; Yang et al., 2025a).

The exploration of FMT in autoimmune diseases presents a heterogeneous clinical picture, reflecting the diverse pathophysiology of these conditions. In a randomized trial for moderate-to-severe atopic dermatitis (AD), FMT significantly improved disease severity scores compared to placebo, which was associated with reduced Th2/Th17 cell proportions and serum IgE, alongside alterations in gut microbiota composition and function (Liu et al., 2025). For systemic lupus erythematosus (SLE), an analysis of patients from a clinical trial suggested that FMT responders exhibited an increase in global DNA methylation, potentially reversing disease-associated hypomethylation patterns, with changes linked to specific microbial metabolites like hexanoic acid (Zhang et al., 2023). This aligns with the broader concept that FMT may restore immune homeostasis in autoimmune diseases by rebuilding the intestinal microecosystem and modulating innate and adaptive immunity (Liu et al., 2023b; Yang et al., 2023). However, results are not uniformly positive. A pilot RCT in active peripheral psoriatic arthritis (PsA) found that a single gastroscopic-guided FMT was inferior to sham transplantation, with a higher rate of treatment failure and less improvement in disability scores in the FMT group (Kragsnaes et al., 2021). This highlights that the efficacy of FMT can be disease- and protocol-specific. A particularly compelling application is in the setting of allogeneic hematopoietic cell transplantation (allo-HCT), where steroid-refractory gastrointestinal acute graft-versus-host disease (GVHD) is a life-threatening complication driven by dysbiosis. Prospective studies have reported that donor FMT can induce complete clinical responses in a majority of such patients, accompanied by increased microbial diversity, engraftment of butyrate-producing bacteria, and successful tapering of immunosuppressants (Van et al., 2020; Henig et al., 2021). This therapeutic effect is thought to stem from restoration of microbial balance and SCFA production, which enhances regulatory T-cell responses and reduces inflammation (Soleimani et al., 2025; Yang et al., 2025b). Early pilot data also suggest potential efficacy for refractory chronic GVHD (Yang et al., 2025b). Furthermore, in immunocompromised patients, FMT has demonstrated effectiveness and safety in decolonizing the gut of MDROs, a critical goal for preventing invasive infections (Bilinski et al., 2017; Battipaglia et al., 2019; Merrick et al., 2025). This decolonization is more likely when the donor and recipient microbiota are dissimilar at baseline and when donor strain engraftment is higher (Merrick et al., 2025).

The field of oncology represents one of the most dynamic areas for FMT research, primarily focused on modulating response to immunotherapy. The gut microbiome significantly influences the efficacy and toxicity of anticancer therapies, including immune checkpoint inhibitors (ICIs) (Wu et al., 2019; Huang et al., 2022b; Bolte et al., 2025). Consequently, FMT is being investigated to overcome primary or acquired resistance to ICIs. A phase I trial in advanced melanoma patients combined FMT from healthy donors with anti-PD1 therapy as first-line treatment, reporting a high objective response rate and promising long-term progression-free and overall survival compared to historical data (Hadi et al., 2025). The integration of FMT with ICIs aims to reshape the tumor microenvironment through modulation of gut microbiome composition and microbial metabolite profiles, though outcomes across studies are variable and context-dependent (Lin et al., 2025). A systematic review noted that while most studies demonstrate the safety and feasibility of FMT in cancer patients, its efficacy in improving treatment outcomes and preventing immune-related adverse events requires confirmation in large-scale RCTs (Wekking et al., 2025). Mechanistic insights from preclinical models are informing this approach. In mice with colitis-associated colorectal cancer, FMT from healthy donors inhibited tumor progression and inflammatory pathways, whereas FMT from CRC patients promoted it, directly linking donor microbiota composition to oncogenic outcomes (Song et al., 2024). FMT improved anti-PD-1 efficacy and progression-free survival in a single-arm trial in advanced PD-L1-negative non-small-cell lung cancer (Chen et al., 2026). Strains from different evolutionary lineages within the same species had opposite treatment effects, thus addressing the inconsistent results in previous related studies (Chen et al., 2026). Beyond immunotherapy, FMT is explored for managing complications like immunotherapy-induced colitis and for its role in the broader context of hematopoietic cell transplantation (Henig et al., 2021; Wekking et al., 2025). The concept of utilizing bacteria or their products directly as therapeutics, including via FMT, is evolving into the field of bacteria-mediated cancer therapy (Jayaprakash et al., 2025).

The expansion of FMT into these diverse systemic diseases underscores both its therapeutic potential and the formidable challenges ahead. The variable clinical responses observed across and within disease categories emphasize that FMT is not a uniform intervention but one whose outcome is dictated by a triad of factors: the donor microbial ecosystem, the recipient’s baseline microbiota and disease state, and the administration protocol (Gulati et al., 2023; Lin et al., 2025). The field is progressively moving from undefined fecal suspensions toward more refined and targeted approaches, such as selecting donors based on specific microbial or metabolic profiles (Ebadi et al., 2025), using washed microbiota (Zhong et al., 2024), or exploring fecal filtrate transplantation to eliminate bacterial cells while transferring bacteriophages and metabolites, as shown in a necrotizing enterocolitis model (Brunse et al., 2022). As research delineates the specific mechanisms—be it through SCFA production, bile acid metabolism, modulation of specific immune cell populations, or bacterial metabolite synthesis—the ultimate goal is the rational design of next-generation, precision microbiota-based therapeutics tailored to correct the distinct dysbiotic networks underlying each extra-intestinal disorder.

## 6 Technical evolution and next-generation therapies: from donor selection to microbial ecologies

The variability in clinical efficacy observed across different diseases and even among patients with the same condition has driven a fundamental evolution in the field of microbiota-based therapies. This evolution aims to move beyond the undefined, complex mixture of a donor’s fecal matter toward more refined, controllable, and targeted interventions. This progression begins at the very source: the donor. A donor-centric administration model is vital for the feasibility, safety, and ethical integrity of stool donor programs (Kabage et al., 2025). The establishment of standardized stool banks, guided by international consensus, has been instrumental in transforming FMT from an *ad hoc* procedure into a more reliable therapy, primarily for rCDI (Cammarota et al., 2019). These banks implement rigorous, multi-step screening protocols for pathogens, including MDROs and viruses, which is especially critical when treating immunocompromised recipients (Ghani et al., 2022). However, donor variability remains a significant challenge, influencing the composition and functional potential of the transplanted microbiota, which in turn impacts engraftment and clinical outcomes (Ghani et al., 2024; Herman et al., 2025).

To mitigate risks and enhance consistency, significant advancements have been made in the processing and preparation of fecal material (**Table 1**). The manual preparation of crude fecal suspensions presents safety risks and psychological barriers (Zhang et al., 2020). WMT not only allows for precise dosing based on microbial cell count rather than stool weight but also significantly reduces adverse events by removing pro-inflammatory metabolites, eukaryotic viruses, and potential toxins (Zhang et al., 2020). Furthermore, maintaining bacterial viability is paramount. Studies demonstrate that processing stool in ambient air, compared to strict anaerobic conditions, drastically reduces the viability of key anaerobic commensals like *Faecalibacterium prausnitzii* and diminishes the capacity for SCFA production, underscoring the importance of oxygen-free processing protocols to preserve the therapeutic potential of the graft (Papanicolas et al., 2019).

Parallel to processing improvements, the routes of administration have diversified to improve patient accessibility, compliance, and target specificity. While colonoscopy and enema are effective lower gastrointestinal routes, they are invasive (Gulati et al., 2020). The development of oral encapsulated formulations has been a breakthrough, enabling non-invasive delivery. Initial studies using third-party FMT capsules after allogeneic hematopoietic cell transplantation demonstrated feasibility and safety, with successful engraftment of donor taxa (DeFilipp et al., 2018). However, challenges such as high capsule burden, gastric acid degradation, and palatability persist (Kamath et al., 2025). Innovative delivery technologies are emerging to address these issues. For instance, encapsulating individual microbial cells or entire communities within protective nanocapsules, such as those made from silk fibroin and phosphatidylcholine, can significantly enhance survival through the gastrointestinal tract and has shown improved efficacy in animal models of colitis compared to conventional FMT (Hou et al., 2025b). Lyophilization (freeze-drying) is another key processing technology that stabilizes microbial communities for long-term storage and enables formulation into a lower volume of capsules, reducing the burden on patients (Kamath et al., 2025).

A profound shift in understanding has been the recognition that therapeutic effects are mediated not only by bacteria but by the entire microbial ecology, including bacteriophages (phages) and fungi. Metagenomic analyses reveal that successful FMT involves the transfer and engraftment of donor-derived phages, which can modulate bacterial populations and metabolic functions in the recipient (Fujimoto et al., 2021; Ji et al., 2025). In rCDI, the transfer of Caudovirales phages is associated with treatment success (Liu et al., 2023a). This has spurred interest in fecal virome transplantation (FVT), which involves administering a sterile filtrate of donor feces containing viruses and metabolites but no live bacteria, thereby eliminating the risk of bacterial pathogen transmission (Rasmussen et al., 2020, Rasmussen et al., 2024). Preclinical studies show that FVT can be as effective as FMT in treating CDI in mice, and further processing steps like solvent/detergent treatment can inactivate enveloped eukaryotic viruses, enhancing safety (Rasmussen et al., 2024). The gut mycobiome, particularly fungi like *Candida* species, also undergoes shifts post-FMT and may contribute to outcomes, though its role is less defined (Lam et al., 2022).

Driven by the desire for greater precision, safety, and regulatory clarity, the field is actively developing next-generation therapies that move beyond whole stool. LBPs are defined consortia of bacterial strains. An example is VE303, a consortium of eight clonal *Clostridia* strains, which in phase 1 studies demonstrated safety, colonization in healthy volunteers after vancomycin pretreatment, and promotion of a microbiota structure associated with colonization resistance (Dsouza et al., 2022). LBPs offer a standardized, pharmaceutical-grade alternative to FMT (Lavoie et al., 2023; Kim et al., 2024). Another innovative approach involves engineering the donor microbiota itself before transplantation. Preconditioning donor mice with a specific probiotic (*Lactiplantibacillus plantarum* GR-4) created a “modified” FMT with enhanced stability, butyrate production, and immunomodulatory metabolite synthesis, leading to superior outcomes in a colitis model compared to conventional FMT (Fan et al., 2026). Beyond live bacteria, research is exploring the therapeutic potential of bacterial derivatives. Extracellular vesicles derived from the gut microbiota, particularly from microbiota “trained” by nanomedicine, have shown efficacy surpassing that of FMT in treating UC in mice by modulating inflammation and metabolism (Zu et al., 2024). Similarly, the concept of selective microbiota transplantation, which focuses on enriching or isolating specific functional groups of microbes, represents a strategic modernization of the crude FMT approach (Zhang et al., 2018).

Finally, the paradigm is expanding to include autologous microbiota transplantation. In patients undergoing intensive antibiotic therapy, such as in allogeneic hematopoietic stem cell transplantation, banking one’s own stool before treatment for later autologous FMT can effectively reconstitute the individual’s baseline microbiota, restoring lost diversity and potentially protecting against complications (Taur et al., 2018). This personalized approach eliminates donor-related variability and safety concerns entirely.

This technical evolution, from meticulous donor selection to the engineering of microbial ecologies and the development of defined biological products, reflects the field’s maturation. It aims to replace the unpredictable “black box” of donor stool with safer, more consistent, and mechanistically driven therapies. As these technologies advance, they must be integrated within robust ethical and regulatory frameworks that balance innovation with patient safety, ensuring that microbiota-based therapies can be effectively and responsibly translated into widespread clinical practice (Kabage et al., 2025; Maor et al., 2025).

## 7 Safety, ethics, and regulatory frameworks: navigating the path to clinical integration

The maturation of microbiota-based therapies, from donor-derived fecal microbiota transplantation (FMT) to engineered microbial consortia, necessitates their integration within robust safety, ethical, and regulatory frameworks to responsibly translate scientific innovation into widespread clinical practice (Yadegar et al., 2024; Maor et al., 2025). While FMT for recurrent *Clostridioides difficile* infection (rCDI) has demonstrated high efficacy, its broader application, particularly in non-CDI indications, must be rigorously balanced against potential risks (Marrs and Walter, 2021; Gulati et al., 2023; Yadegar et al., 2024). Safety considerations are paramount. Short-term adverse events, such as abdominal discomfort, diarrhea, and bloating, are typically mild and self-limiting (Yau et al., 2024). However, serious adverse events, including the transmission of enteric pathogens or MDROs due to inadequate donor screening, have been documented, underscoring the critical need for stringent donor selection and stool processing protocols (Ghani et al., 2022; Mullish et al., 2024). Prospective long-term registry data, such as that from Hong Kong spanning over eight years, provide reassuring evidence, indicating a low risk of developing new medical conditions (e.g., inflammatory bowel disease, metabolic disorders) beyond 12 months post-FMT that can be attributed to the procedure itself (Yau et al., 2024). Nevertheless, the rise of the Do-It-Yourself (DIY) FMT movement, largely facilitated by online information, presents a distinct public health challenge (Ekekezie et al., 2020). Surveys indicate individuals are performing DIY-FMT for conditions like inflammatory bowel disease and irritable bowel syndrome with little evidence of efficacy, often using donors screened only by personal acquaintance, thereby circumventing established safety protocols and exposing themselves to significant, unmonitored risks (Ekekezie et al., 2020).

These safety concerns are deeply intertwined with complex ethical issues that must be addressed to protect patients and donors (Ma et al., 2017). The vulnerability of patients with debilitating or refractory conditions creates a potential for exploitation, where desperation may drive acceptance of unproven treatments without fully appreciating the risks (Ma et al., 2017). A robust informed consent process is therefore essential, requiring clear communication about the investigational nature of FMT for most indications, the incomplete understanding of long-term consequences, and the potential for unknown risks (Ma et al., 2017; Ng et al., 2020). Defining a “suitable healthy donor” is another ethical cornerstone, extending beyond the absence of infectious diseases to considerations of lifestyle, medication use, and potential risk factors for chronic diseases, a process that must respect donor autonomy and confidentiality (Ma et al., 2017; Cammarota et al., 2019). Furthermore, ensuring equitable access to FMT while preventing its premature commercialization and unethical promotion is a significant challenge for healthcare systems (Ma et al., 2017). This is particularly relevant given that many patients with rCDI referred for FMT, especially those who are immunocompromised or have inflammatory bowel disease, are systematically excluded from industry-sponsored RCTs of novel microbiome therapeutics, raising questions about the generalizability of trial results and access to potentially life-saving treatments (Kelly et al., 2020).

The regulatory landscape for live biotherapeutic products, including FMT, is evolving to address these safety and ethical imperatives while fostering innovation (Maor et al., 2025). A proposed conceptual framework, the “Ladder of Regulatory Stringency and Balance,” suggests that regulations should be proportionate to the risks posed by specific products within a technological class (Maor et al., 2025). For donor-derived FMT used in rCDI, this analysis positions it close to, but below, a threshold for under-regulation, recommending enhanced oversight of donor screening, processing, and distribution to ensure regulatory balance (Maor et al., 2025). Achieving this requires comprehensive standardization, an area historically lacking. A systematic review of FMT study reporting found that critical methodological components—such as donor eligibility criteria, stool processing and conservation methods, and the amount of material administered—were frequently omitted from publications, hindering replication and a clear understanding of safety and efficacy signals (Bafeta et al., 2017). This reporting deficiency extends to preclinical studies, prompting the development of Guidelines for Reporting Animal Fecal Transplant (GRAFT) to improve the rigor and translatability of animal model research (Secombe et al., 2021).

In response, international and national consensus conferences have worked to establish best practices for FMT in clinical care. Key recommendations center on the establishment of dedicated FMT centers or stool banks, which are seen as crucial for ensuring reliable, safe, and equitable access to quality-controlled products (Cammarota et al., 2017, Cammarota et al., 2019; Mullish et al., 2024). These entities provide a structured framework for implementing rigorous, multi-tiered donor screening protocols that encompass medical history, behavioral risk assessments, and extensive blood and stool testing for pathogens, including MDROs and viruses like SARS-CoV-2 (Cammarota et al., 2019; Haifer et al., 2020; Ianiro et al., 2020; Ghani et al., 2022). The COVID-19 pandemic necessitated specific adaptations to these protocols, reinforcing the need for dynamic screening criteria that respond to emerging public health threats (Ianiro et al., 2020). Consensus guidelines, such as those from the British Society of Gastroenterology, also provide clear recommendations on clinical indications, administration routes, and monitoring, distinguishing between established use in rCDI and investigational use in other conditions (Mullish et al., 2024). Similarly, expert consensus in China has aimed to standardize clinical application and management amid evolving regulatory landscapes (National Institute of Hospital Administration et al., 2022).

Looking ahead, the regulatory pathway will need to adapt to the next generation of microbiome-based therapies, including defined microbial consortia and engineered live biotherapeutic products (Ng et al., 2020; Maor et al., 2025). These products, with their more standardized and controllable compositions, may fit more readily into traditional drug regulatory paradigms compared to donor-derived FMT (Yadegar et al., 2024). Regardless of the product type, ongoing post-marketing surveillance and long-term registries will be indispensable for detecting rare or delayed adverse events, understanding the durability of therapeutic effects, and refining patient selection criteria (Cammarota et al., 2019; Yau et al., 2024). The journey from a complex, donor-dependent procedure to a suite of precision microbiome therapies hinges on navigating this intricate interplay of demonstrated safety, ethical rigor, and adaptive, proportionate regulation.

## 8 Conclusion and future perspectives

The trajectory of FMT reflects a broader paradigm shift in biomedicine, evolving from an empirical procedure to a foundational platform for developing next-generation, precision microbiome-based therapeutics (Yadegar et al., 2024; Wang et al., 2025c). While FMT has decisively established itself for rCDI, its investigation across a spectrum of gastrointestinal, metabolic, neurological, and oncological disorders has illuminated both the profound therapeutic potential and the inherent complexities of modulating the human ecosystem (Wang et al., 2022; Andary et al., 2024). The future of this field lies not in the indiscriminate transfer of complex fecal matter but in the rational design and targeted application of microbial interventions tailored to the specific dysbiotic networks and pathophysiological needs of individual patients (Andary et al., 2024; Nagayama et al., 2025). This transition from a “one-size-fits-all” approach to personalized microbiota-based medicine is the central challenge and opportunity that will define the next decade of research and clinical translation.

Achieving this vision of precision requires a multi-faceted strategy. It necessitates moving beyond descriptive taxonomic analyses of dysbiosis toward a functional understanding of disturbed microbial networks and their metabolic outputs in specific disease contexts (Andary et al., 2024). Success will depend on refining donor-recipient matching criteria, potentially based on deep microbial phenotyping, immune profiles, and metabolomic signatures, rather than relying solely on general health status (Lin et al., 2025; Nagayama et al., 2025). For conditions like IBD or cancer immunotherapy, where host-microbiome-immune interactions are paramount, the efficacy of interventions may be maximized by selecting donors whose microbiota exhibits functional traits known to support favorable immune modulation or therapeutic response (Jamal et al., 2023; Lin et al., 2025). Furthermore, personalization extends to administration protocols, considering factors such as disease stage, prior treatments, and individual gut ecology to optimize engraftment and durability of effect (Hou et al., 2025a; Wang et al., 2025c).

Technologically, the field is rapidly diversifying beyond donor-derived FMT. The development of defined LBPs and rationally designed microbial consortia represents a critical step toward standardization, safety, and regulatory approval (Kim et al., 2024; Yadegar et al., 2024). These products aim to recapitulate or enhance the therapeutic functions of a healthy microbiota using a controlled set of well-characterized bacterial strains (Quiroga-Centeno et al., 2025). Concurrently, advanced bioengineering is enabling the creation of engineered live biotherapeutics that can perform specific functions, such as delivering anti-inflammatory molecules directly to the gut mucosa in IBD or producing immunomodulatory metabolites to enhance cancer immunotherapy (Quiroga-Centeno et al., 2025; Li et al., 2026). Another frontier is the integration of microbiome modulation with nanomedicine, where nanomaterials could be used to precisely deliver microbial consortia or target and eliminate pathogenic elements of the microbiota within the complex tumor microenvironment (Huang et al., 2025). The integration of multi-omics data (metagenomics, metatranscriptomics, metabolomics) with artificial intelligence and machine learning will be indispensable for deciphering complex host-microbe interactions, predicting treatment responses, and designing these next-generation therapies (Lin et al., 2025; Li et al., 2026).

Despite the exciting prospects, significant challenges must be navigated. The long-term safety profile of microbiota manipulations, especially in immunocompromised or elderly populations, requires rigorous and sustained evaluation (Lista et al., 2025; Novelle et al., 2025). The dynamic nature of the gut microbiome across the human lifespan, from infancy to old age, adds a layer of complexity, suggesting that effective interventions may need to be life-stage-specific (Schoultz et al., 2025). Regulatory frameworks must continue to evolve to accommodate these novel therapeutic modalities, which straddle the boundaries between tissue products, biologics, and drugs (Yadegar et al., 2024; Sharma et al., 2025). Ethical considerations regarding donor screening, informed consent for experimental applications, and equitable access remain paramount (Lin et al., 2025). Furthermore, the field must address the inconsistency in clinical trial outcomes for many indications, which underscores the need for larger, well-designed, multi-center RCTs with standardized protocols and rigorous biomarkers of efficacy (Lin et al., 2025; Lista et al., 2025; Girdhar et al., 2026).

Looking forward, the scope of personalized microbiota-based medicine is vast. Beyond established areas, compelling preclinical and early clinical data suggest potential applications in promoting healthy aging by transferring a “youthful” or rejuvenated microbiota (Novelle et al., 2025), in modulating the progression of neurodegenerative disorders like Alzheimer’s disease via the gut-brain axis (Lista et al., 2025; Sharma et al., 2025), and in primary prevention or modulation of autoimmune conditions such as type 1 diabetes (Girdhar et al., 2026). In oncology, the strategic modulation of the microbiome to overcome resistance to immune checkpoint inhibitors represents a paradigm shift toward combinatorial immuno-microbiota therapy (Jamal et al., 2023; Li et al., 2026). The systematic review documenting FMT’s exploration in 85 different diseases is a testament to the breadth of its perceived potential (Wang et al., 2022). Ultimately, the goal is to develop a versatile toolkit of microbiome-targeting strategies—ranging from refined FMT protocols and LBPs to engineered microbes and phage cocktails—that can be deployed with precision based on a patient’s unique microbial and disease fingerprint. This journey from the chaos of whole-stool transplantation to the order of precision microbial ecologies holds the promise of ushering in a new era of therapeutic innovation that addresses the root cause of numerous dysbiosis-associated diseases.
